# Effectiveness and safety of Tiaogan formula in the treatment of coronary heart disease: A protocol for systematic review and meta-analysis

**DOI:** 10.1097/MD.0000000000032237

**Published:** 2022-12-16

**Authors:** Chen Mingtai, Zhong Guofu, Chen Junteng, Men Ling, Luan Jienan, Luo Zhihao

**Affiliations:** a Department of Cardiovascular Disease, Shenzhen Traditional Chinese Medicine Hospital, Shenzhen, China; b Intensive Care Unit, Shenzhen Traditional Chinese Medicine Hospital, Guangzhou University of Chinese Medicine, Shenzhen, China; c Nephrology Department, Shenzhen Traditional Chinese Medicine Hospital, Shenzhen, China; d Department of Emergency Center, Hainan Traditional Chinese Medicine Hospital, Hainan, China.

**Keywords:** coronary heart disease, meta-analysis, protocol, randomized trial, Tiaogan formula

## Abstract

The morbidity and mortality of coronary heart disease (CHD) has remained high, which greatly increases people’s economic burden. Several studies have showed that Tiaogan formula (TGF), as a kind of Chinese herbal medicine, was of benefit to relieving angina pectoris symptoms and improving the quality of life for CHD patients. However, the intensity of evidence has been poor, limiting the further clinical application of TGF to CHD. This systematic review and meta-analysis will assess the effectiveness and safety of studies of TGF in CHD patients.

**Methods:**

A systematic search for literature up to December 2022 will be conducted in following public electronic databases: PubMed, Embase, the Cochrane Library, China National Knowledge Infrastructure, Chinese Scientific Journals Database Database, and Wanfang Database. Inclusion criteria are randomized controlled trials of Tiaogan formula in the treatment of coronary heart disease. The primary outcome measures will be mortality, acute cardiovascular events, total efficacy rate, and improvement of angina symptoms. The secondary outcome measures will be electrocardiogram, levels of blood lipid, and adverse events. RevMan 5.4 software Cochrane Collaboration (London, United Kingdom) will be applied for data synthesis, sensitivity analysis, subgroup analysis, and risk of bias assessment. A funnel plot will be developed to evaluate reporting bias and Egger tests will be used to assess funnel plot symmetries. We will use the Grading of Recommendations Assessment, Development and Evaluation system to assess the quality of evidence.

**Results:**

This study will provide a systematic review of Tiaogan formula in the treatment of CHD.

**Conclusion:**

This study will provide a high-quality synthesis of the effects and safety of Tiaogan formula in the treatment of CHD patients.

## 1. Introduction

It has been estimated that cardiovascular diseases, principally coronary heart disease (CHD) and stroke, are the leading cause of global mortality and a major contributor to disability.^[1]^ The morbidity and mortality of CHD has remained high, which greatly increases people’s economic burden.^[2,3]^ Several factors involving inflammatory response, abnormal lipid, amino acid, carbohydrate and bile metabolism can exacerbate the pathological development of CHD.^[4–6]^ In the early stage of CHD, coronary atherosclerosis is the initial pathology which is characterized by lipid deposition and chronic inflammatory response. Not only can CHD increase mortality, but also severely influence patients’ daily activities and quality of life.^[7,8]^ Current conventional therapies (revascularization, western medication) have been utilized in the treatment on CHD, however, there have been plenty of CHD patients impotent to alleviate angina related symptoms totally and accompanied by adverse effects like dizziness and headache.^[9–11]^ As far as the disadvantages of conventional therapies are concerned, Chinese herbal medicine, which has been applied for thousands of years, may provide a potential complementary treatment strategy for CHD.

According to the basic theories of traditional Chinese medicine (TCM), CHD belongs to the category of “Xiong Bi (chest pain or heartache),” recorded in the TCM classic “Huang Di Nei Jing” since Western Han Dynasty.^[12]^ The etiology and pathogenesis of CHD are related to blood stasis and qi stagnation, which blocks blood circulation of the body. The movement of blood and qi in the body is strongly associated with the function of “liver (gan)” based on the TCM theory.^[13–16]^ Therefore, the TCM therapy principle of “Tiaogan (improving the liver function)” has been applied to regulating qi stagnation and activating blood circulation commonly.^[15,16]^ Xuefu zhuyu decoction, one of the representatives of “Tiaogan” formulas, has been utilized to treat CHD and originated from “Yi Lin Gai Cuo” since Qing Dynasty.^[17,18]^ Currently, more and more evidences prompt that Tiaogan formula (TGF) is of benefit to relieving angina pectoris symptoms and improving the quality of life for CHD patients.^[19–23]^

Although increased numbers of clinical studies and reviews about TGF applied on CHD patients, the intensity of evidence has not been strong enough and there has been lack of meta-analysis to assess the effectiveness and safety of TGF in the treatment of CHD systematically. In virtue of the lack of systematic review about this crucial issue, this meta-analysis will be designed to systematically evaluate the effectiveness and safety of TGF in the treatment of CHD patients.

## 2. Methods and analysis

### 2.1. Registration

This protocol has been registered on international prospective register of systematic review. The trial registration number of prospective register of systematic review is CRD 42018094538. The procedure of this protocol will be conducted according to the Preferred Reporting Item for Systematic Review and Meta-analysis Protocols guidance.^[24]^

### 2.2. Eligibility criteria

#### 2.2..1. Type of study.

We will include all the randomized controlled trials that study the effectiveness and safety of TGF combined with conventional medication in the treatment of CHD.

#### 2.2..2. Participants.

The study will include patients diagnosed as CHD regardless of their age, sex, ethnicity, education or economic status and whether or not they were outpatients or inpatients. The diagnostic criteria of stable angina pectoris are as follows.

The diagnostic criteria of CHD should be confirmed according to one of the past or current definitions. The diagnostic criteria include “Nomenclature and criteria for diagnosis of ischemic heart disease”^[25]^ or “ACC/AHA 2002 guideline update for the management of patients with chronic stable angina task force on practice guidelines (committee to update the 1999 guidelines).”^[26]^

### 2.3. Interventions

Interventions involving the combination of TGF with conventional medication are eligible in intervention group. The same conventional medication must be used in the control group.

### 2.4. Outcome

The primary outcome measures will include: mortality, acute cardiovascular events, total efficacy rate, improvement of angina symptoms, and adverse events. The secondary outcome measures will include: changes of electrocardiogram, levels of total cholesterol, triglyceride, low-density lipoprotein cholesterol and high-density lipoprotein cholesterol levels, and TCM syndrome score.

### 2.5. Search strategy

The following electronic bibliographic databases will be retrieved from inception to December 2022: PubMed, Embase, the Cochrane Library, China National Knowledge Infrastructure, Chinese Scientific Journals Database Database and Wanfang Database. There are no limits on the language of publication. Only clinical trials as a limitation will be included and searched. The following sources will also be searched to identify clinical trials which are in progress or completed: Clinical Trials.gov and World Health Organization clinical trials registry. The additional relevant studies will also be retrieved from the reference lists of systematic reviews and included studies. We will map search terms to controlled vocabulary if possible. In addition, the search strategy for selecting the fields of title, abstract or keyword will be different referring to the characteristics of databases. Search terms are grouped into 3 blocks (see Table 1).

**Table 1:** Search items.

Search block	Search items
Participants	Angina Pectoris OR Anginas, Stable OR Stable Angina OR Stable Anginas OR Chronic Stable Angina OR Angina, Chronic Stable OR Anginas, Chronic Stable OR Chronic Stable Anginas OR Stable Angina, Chronic OR Stable Anginas, Chronic OR Angina Pectoris, Stable OR Angina Pectori, Stable OR Pectori, Stable Angina OR Pectoris, Stable Angina OR Stable Angina Pectori OR Stable Angina Pectoris OR Stenocardia OR Stenocardias OR Angor Pectoris OR Coronary Diseases OR Disease, Coronary OR Diseases, Coronary OR Coronary Heart Disease OR Coronary Heart Diseases OR Disease, Coronary Heart OR Diseases, Coronary Heart OR Heart Disease, Coronary OR Heart Diseases, Coronary
Intervention	Tiaogan formula OR Tiaogan OR Tiaogan therapy OR Tiaogan prescription OR TGF OR Drugs, Chinese Herbal OR Chinese Drugs, Plant OR Chinese Herbal Drugs OR Herbal Drugs, Chinese OR Plant Extracts, Chinese OR Chinese Plant Extracts OR Extracts, Chinese Plant OR Medicine, Chinese Traditional OR Traditional Chinese Medicine OR Chung I Hsueh OR Hsueh, Chung I OR Traditional Medicine, Chinese OR Zhong Yi Xue OR Chinese Traditional Medicine OR Chinese Medicine, Traditional
Study design	Randomized controlled trial OR controlled clinical trial OR randomized OR placebo OR drug therapy OR randomly OR trial OR groups

### 2.6. Study selection and data extraction

**Figure 1.**
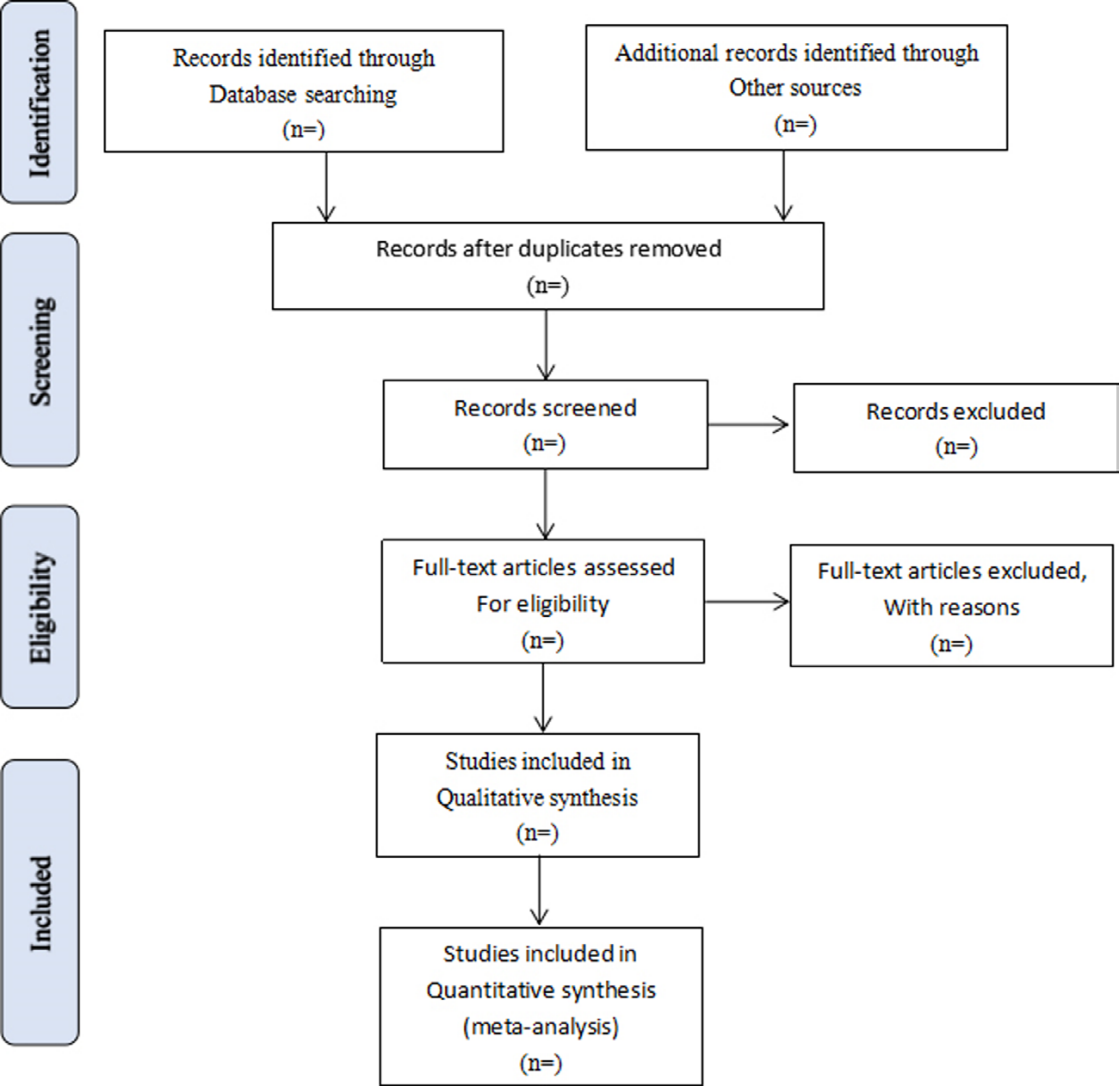
Flow diagram of study selection process. PubMed, Embase, the Cochrane Library, China National Knowledge Infrastructure, Chinese Scientific Journals Database Database, and Wanfang Database.

Literature retrieved citations will be managed by EndNote X7 software (Clarivate Analytics, London, United Kingdom). Two authors (CM and ZG) will screen the titles and abstracts of the all studies retrieved in above electronic databases independently to find potentially eligible studies. Articles which are duplicated or not accordant with eligibility criteria, intervention and outcome in this study will be excluded. After filtering the final eligible articles, the data from the included articles will be extracted independently from 2 authors (CM and ZG). Disagreements will be resolved by discussion or arbitrated by a third author if needed. The following data items will be extracted: first author, publication year, diagnosis information, age, sex, trial characteristics, interventions and controls, participants, study methodology, outcomes, adverse events and etc. (see Fig. 1).

### 2.7. Risk of bias assessment

The methodological quality of the eligible studies will be evaluated according to the Cochrane Collaboration’s tool is included in RevMan 5.4 software for assessing risk of bias.^[27]^ The assessment details include: sequence generation, allocation concealment, blinding of participants and personnel, blinding of outcome assessors, incomplete outcome data, selective reporting and other sources of bias. Each domain will be assessed as “low risk” or “high risk” or “unclear risk” according to the description details of eligible studies.

### 2.8. Data synthesis and statistical analysis

Statistical analyses will be conducted with RevMan 5.4 software provided by Cochrane Collaboration. Data will be presented by risk ratio or odd ratio with its 95% confidence interval for dichotomous outcomes and standardized mean difference or weighted mean difference with its 95% confidence interval for continuous outcomes. The *I*^2^ test will be calculated to determine the amount of heterogeneity. The results of the studies could be used the fixed-effect model to combined unless *I*^2^ statistic is >50%, in which cases, the random-effects model will be used.

### 2.9. Sensitivity analysis, subgroup analysis, and meta-regression

If the heterogeneity or inconsistency among the studies was detected, sensitivity analysis or subgroup analysis or meta-regression analysis will be performed. Subgroup analysis will be conducted to explore potential sources of heterogeneity according to the characteristics of studies, including sample size, severity of angina, dose of Chinese herbal medicine formulas, treatment duration and other relevant parameters. If data extraction is insufficient, we will create a qualitative synthesis.

### 2.10. Publication bias

A funnel plot will be developed to evaluate reporting bias of the included studies. We will use Egger tests to assess funnel plot symmetry and will interpret values of *P* < .1 as showing statistical significance.

### 2.11. Quality of evidence

We will also assess the quality of evidence for the main outcomes with the Grading of Recommendations Assessment, Development and Evaluation approach. The 5 items will be investigated, including limitations in study design, inconsistency, inaccuracies, indirectness and publication bias.

### 2.12. Patient and public involvement

Patients and/or public were not involved due to this study belonging to the secondary sources analysis.

## 3. Discussion

It has been known that TGF has been applied to treating CHD for a long time.^[16,17]^ Nowadays, TGF is commonly and widely applied as a complementary treatment strategy for CHD patients in China. Previous studies have indicated that TGF contributes to downregulating levels of blood lipid, relieving angina pectoris and improving the electrocardiogram for CHD patients.^[20–23]^ Increased numbers of clinical studies prompted that TGF has been widely used and beneficial in improving clinical indicators for CHD patients, however, there has been no comprehensive assessment of the clinical evidence regarding TGF as intervention for CHD in evidence-based medicine.^[20–23]^ Consequently, this meta-analysis intends to assess the effectiveness and safety of TGF for CHD patients. The results of this meta-analysis may provide more appropriate and objective clinical recommendation for clinician to apply TGF therapy to CHD patients.

## Author contributions

Zhihao Luo and Mingtai Chen conceived the study and drafted the protocol. Jienan Luan and Guofu Zhong revised it. Mingtai Chen, Ling Men, Junteng Chen and Guofu Zhong developed the search strategies, will conduct data collection, and analyze the data independently.

**Conceptualization:** Mingtai Chen, Zhihao Luo.

**Data curation:** Guofu Zhong.

**Formal analysis:** Guofu Zhong.

**Funding acquisition:** Mingtai Chen, Jienan Luan.

**Investigation:** Mingtai Chen.

**Methodology:** Mingtai Chen.

**Project administration:** Mingtai Chen, Jienan Luan.

**Resources:** Ling Men, Jienan Luan.

**Software:** Ling Men.

**Supervision:** Jienan Luan.

**Validation:** Junteng Chen.

**Visualization:** Junteng Chen.

**Writing – review & editing:** Zhihao Luo.
